# *Spätzle* Regulates Developmental and Immune Trade-Offs Induced by *Bacillus thuringiensis* Priming in *Rhynchophorus ferrugineus*

**DOI:** 10.3390/insects15120925

**Published:** 2024-11-26

**Authors:** Pengju Li, He Zhang, Anran Tan, Zhuolin Hu, Lu Peng, Youming Hou

**Affiliations:** 1 State Key Laboratory of Ecological Pest Control for Fujian and Taiwan Crops, Fujian Agriculture and Forestry University, Fuzhou 350002, China; big_fat_ju@126.com (P.L.); zhhe1205@163.com (H.Z.); 18120967860@163.com (A.T.); m18396513018@163.com (Z.H.); 2 Key Lab of Biopesticide and Chemical Biology, Ministry of Education & Fujian, Fujian Agriculture and Forestry University, Fuzhou 350002, China; 3 Province Key Laboratory of Insect Ecology, College of Plant Protection, Fujian Agriculture and Forestry University, Fuzhou 350002, China

**Keywords:** red palm weevil, *Bacillus thuringiensis*, immune priming, *Spätzle*, fitness cost

## Abstract

The red palm weevil (RPW) is an invasive pest that causes devastating damage to a variety of palm plants, which exhibit specific immune priming to *Bacillus thuringiensis* (Bt). However, immune priming in RPW may incur a high fitness cost, and its molecular signaling pathways have not yet been reported. Here, we investigated the effect of Bt priming on RPW development and subsequently analyzed the hormonal and immune-related molecular pathways influencing the fitness cost induced by Bt priming. Bt priming delayed the body weight gain of fifth-instar larvae and prolonged their developmental duration. Bt priming significantly reduced the 20-hydroxyecdysone (20E) content in RPW hemolymph, and the expression levels of the 20E biosynthesis-related genes *SHADOW* and *SHADE* were significantly downregulated. Furthermore, we analyzed Toll pathway genes influencing Bt priming and found that only *Spätzle* (*SPZ*) transcription was significantly activated under Bt priming. After silencing *SPZ* expression, the negative effects of Bt priming on development, *SHADOW* expression, and 20E synthesis were eliminated, thereby suggesting that *SPZ* is a key molecular signal mediating developmental and immune trade-offs induced by Bt priming. Our results elucidate the molecular cascade pathway of immune priming and provide new targets for improving the efficiency of RPW biological controls.

## Simple Summary

Insects have specific immune priming to pathogenic microorganisms, such as *Bacillus thuringiensis* (Bt). However, there may be a trade-off between immunity and development during immune priming and its molecular signaling pathways have not yet been reported. Our study found that Bt priming regulated the development and immune trade-offs in *Rhynchophorus ferrugineus* by activating the immune gene *Spatzle*, thereby inhibiting *SHADOW* transcription and 20E synthesis. Our results contribute to a deeper understanding of the trade-off during immune priming of insects and provide new targets for biological controls of *Rhynchophorus ferrugineus*.

## 1. Introduction

During evolution to adverse environmental stress and pathogen infection, organisms can exhibit two immune defense modes: innate and acquired immunity [1]. Unlike vertebrates, insects are generally considered to lack acquired immunity and rely primarily on innate immunity without immune memory against pathogens [2,3,4,5]; however, recent studies have found that some insects exposed to non-lethal doses of pathogen infestation or external stimuli can initiate a faster and more efficient immune response to increase survival when re-infected, a phenomenon known as immune priming [6,7,8]. Some studies have confirmed the existence of immune priming in certain insect species, including *Tribolium castaneum*, *Bombyx mori*, and *Drosophila melanogaster* [9,10,11]. Shi et al. discovered immune priming in red palm weevils (RPW) [12]. However, immune priming initiated by different pathogens is species-specific [13].

Insects produce a series of immune defensive responses during priming to protect against reinfection. For example, *Bacillus thuringiensis* (Bt)-mediated immune priming caused a significant increase in phenoloxidase (PO) activity in the RPW hemolymph and led to rapid immune resistance upon reinfection [12], as well as an increase in the concentration of hemocytes and phagocytosis while inducing the expression of immune-related genes [7]. The activities of PO and lysozyme in the hemolymph increased and the expression of antimicrobial peptides was induced after the immune priming of *Teleogryllus oceanicus* [14]. Additionally, a clear boost in reactive oxygen production in *Parasemia plantaginis* was observed through Bt-mediated immune priming [15]; however, immune responses induced by immune priming consume considerable energy and affect the allocation of limited resources, thus imposing a certain fitness cost [16].

Genetic and physiological trade-offs exist among fitness-related traits, which is a fundamental principle of life history theory [17,18]. Immune priming in insects evolves in response to different pathogens and continuous stimulation, suggesting phenotypic plasticity in immune priming [19,20,21,22]; however, the emergence of phenotypic plasticity implies a high investment in immune responses; therefore, it must be weighed against other fitness traits, such as development [23]. In response to adverse environments, *Galleria mellonella* can prolong development and reduce growth to improve its immune capacity [24]. Conversely, when *G. mellonella* interacts with parasitic wasps, parasite-induced immune priming prompts the host to activate its immune system, thereby accelerating development [25]. Zanchi et al. found that if adult *Tenebrio molitor* were infected with pathogens, the developmental duration of their offspring was significantly prolonged [26]; however, the opposite result was observed in *T. castaneum* [27]. These results suggest that the fitness trade-offs triggered by immune priming are species-specific.

Hormones are important growth regulators that are crucial for controlling fitness trade-offs through endocrine signaling pathways to maintain the physiological homeostasis of arthropod species [28,29]. In insects, hormones such as the juvenile hormone (JH) and 20-hydroxyecdysone (20E) induce developmental transitions, including molting and metamorphosis; therefore, changes in hormone titers can affect growth and development [30,31], and JH and 20E are considered immunomodulators [32]. These immunomodulators may represent a potential regulatory factor in the balance between insect immunity and development [28,33]. Palli et al. showed that JH titers increased and ecdysteroid titers decreased after the entomopoxvirus infection in *Choristoneura fumiferana*, resulting in developmental delays and mass death of the final larvae [34]. Similarly, the entomopoxvirus infection reduced ecdysone titers and maintained the status quo levels of JH by preventing its metabolism in *Mythimna separata* larvae, thus delaying development [35]; however, the molecular mechanisms and signaling pathways through which hormones regulate immune priming and fitness trade-offs remain unclear.

The RPW, *Rhynchophorus ferrugineus* Olivier, is a highly invasive quarantine pest that poses a serious threat to palm plants in China. RPWs feed within the palm trunks and create crisscross tunnels, which makes their control extremely difficult [36]. Our previous study found that RPW exhibits specific immune priming against *Bacillus thuringiensis* (Bt), which leads to an increase in hemocyte differentiation and phagocytosis as well as an induction of the immune-related gene expression [7,12]. However, the fitness costs associated with immune priming, along with the regulatory mechanisms and molecular signaling pathways influencing these trade-offs, remain unclear. In this study, to address this research gap, we investigated the effects of Bt priming on RPW development. Subsequently, the hormone- and immune-related molecular pathways associated with fitness costs induced by Bt priming were analyzed. This is the first study to explore the trade-off between immune priming and fitness costs from the perspective of the interaction of hormones and immune signaling in RPW, and our results provide new insights and targets for improving the efficiency of RPW biological control.

## 2. Materials and Methods

### 2.1. Insects and Entomopathogen

The RPWs used in this study originated from the palm plantation in Quanzhou and Zhangzhou City, Fujian Province, China, and were reared in the Pest Ecological Control Laboratory, Fujian University of Agriculture and Forestry. The adults and larvae were fed with fresh sugarcane at 28 ± 1 °C, 65 ± 5% relative humidity, and a 14:10 h light:dark (L:D). Pairs of RPW adults were placed in a permeable plastic tank (8 × 11 cm^2^) for rearing. The larvae were reared individually in a plastic box (inner grid size: 19 × 12.7 × 3.5 cm^3^, 24 grids) with a regular diet change [7,37,38]. Fifth-instar larvae (approximately 500 mg) were selected for follow-up tests [7].

*Bacillus thuringiensis* (Bt) was used for the infection and priming, as it demonstrates insecticidal activity against RPW [37,38]. Bt was first electroporated into the PBR322 plasmid (Solarbio, Beijing, China) to attain tetracycline resistance and then cultured on Luria–Bertani (LB) liquid medium on a shaker at 30 °C with 200 rpm until reaching an OD600 of approximately 1.0.

### 2.2. Preparation of Bt Priming Suspension

Bt was centrifuged at 4 °C with 2300× *g*, and the supernatant was removed. Then the tube was washed thrice with PBS buffer. The concentration of Bt suspension was adjusted to 10^8^ cells/mL using a PBS solution and then inactivated by heating at 100 °C for 25 min for immune priming [7]. Part of the Bt suspension was cultured in an LB solid medium to verify the inactivation effect, and the remainder was stored at −80 °C until use.

### 2.3. Effect of Bt Priming on Biological Parameters

The PBS solution (10 μL) with heat-killed cells of Bt (1 × 10^8^) was injected into the hemolymph of fifth-instar larvae for Bt priming, and 10 μL of PBS solution was injected as a control, and the consistent processing was used in subsequent Bt priming. There were 30 fifth-instar larvae in both the treatment and control groups. Body weight was measured daily until molting, and the developmental duration was recorded.

### 2.4. Hormone Content Measurement

The 20E [39], JH [40], and insulin (INS) [41] contents in the RPW hemolymph were determined using an ELISA Kit (Meike, China) according to the manufacturer’s instructions. First, the hemolymph of the fifth-instar larvae (weight 0.50 ± 0.05 g) was extracted at 1, 3, and 6 d after Bt priming; then, the hemolymph, the standard substance, and the HRP-conjugate reagent-labeled detection antibody were successively added to the micropores pre-coated with the 20E, JH, and INS antibody, respectively. After incubation at 37 °C for 60 min and five rounds of washing, the substrate 3,3′,5,5′-Tetramethylbenzidine (TMB) was added for chromogenic reaction. The absorbance (OD value) was measured using a microplate reader at 450 nm. The regression equation of the standard curve was calculated according to the concentration and OD values, and the 20E, JH, and INS contents were calculated [39,40,41]. Each sample consisted of three larvae with five replicates.

### 2.5. RT-qPCR Analysis

Genes in the Toll and 20E synthesis pathways of *D. melanogaster* and *T. castaneum* were downloaded from NCBI GenBank (https://www.ncbi.nlm.nih.gov/) “URL (accessed on 1 May 2022)” [42,43,44] and used as queries against the RPW genome (https://www.ncbi.nlm.nih.gov/assembly/GCA_014462685.1/) “URL (accessed on 1 May 2022)” using the local BLASTP Program (E-value < 10^−5^). For RT-qPCR, total RNA was extracted from the hemolymph of the fifth-instar larvae (weight 0.5 ± 0.05 g) at 1, 3, and 6 d after Bt priming using the Whole Blood Total RNA Extraction Kit (Aidlab, Beijing, China), following the manufacturer’s instructions, and its concentration, purity, and integrity were determined using a NanoDrop 2000 (Thermo Fisher Scientific, Waltham, MA, USA) and agarose gel electrophoresis, respectively. The cDNA template was synthesized using the PrimeScript TM RT Reagent Kit with gDNA Eraser (Takara, Beijing, China) with 1000 ng RNA. RT-qPCR was performed using the Eastep^®^ RT Master Mix kit (Promega, Shanghai, China) with the following procedure: 95 °C for 10 min in pre-denaturation, and 40 cycles of 95 °C for 15 s and 60 °C for 1 min. Each sample was composed of six larvae with a total of three replicates, and the fold change of the expression was calculated using the comparative Ct method (2^−ΔΔCt^). The primers used are listed in Table S1; *GAPDH* was used as a reference gene.

### 2.6. RNA Interference

Specific primers containing the T7 RNA polymerase promoter sequence were designed for use in the double-stranded RNA (dsRNA) synthesis (Table S1). The PCR program was performed as follows: 98 °C for 3 min, 35 cycles of 98 °C for 30 s, 68.3 °C for 30 s, 72 °C for 30 s, and an additional extension at 72 °C for 5 min. PCR products were further purified using a Gel Extraction Kit (Tiangen, Beijing, China). dsRNA of *SPZ* and e*GFP* was synthesized using the HiScribe T7 Quick High Yield RNA Synthesis Kit (New England Biolabs, Ipswich, MA, USA) according to the manufacturer’s protocols [7]. ds*SPZ* (2 μg) was injected into the hemolymph of the fifth-instar larvae with a Nanoliter 2010 Injector (WPI, Sarasota, FL, USA), and ds*eGFP* (2 μg) was injected as a negative control. Hemolymph was collected from the injected individuals 24 h after dsRNA injection to verify RNAi efficiency. The transcription levels of *SHADOW* and *SHADE*, as well as the content of 20E, were also determined using RT-qPCR and ELISA (refer to Section 2.5 and Section 2.4, respectively). Each sample consisted of five larvae with three replicates. The specific primers are listed in Table S1.

### 2.7. Detection of Bt Priming Effects After SPZ Interference

The fifth-instar larvae were injected with 2 μg of ds*SPZ*, followed by a separate injection of 10 μL PBS and heat-killed Bt (10^8^ cells/mL). Larvae were then reared individually in a plastic box (see Section 2.1), and the body weight and developmental duration were recorded (see Section 2.3), which included 15 larvae each in the treatment and control groups. Additionally, the hemolymph of the larvae was collected 24 h after the ds*SPZ* and Bt as well as ds*SPZ* and PBS injections to detect the *SHADOW* expression and 20E synthesis (see Section 2.5 and Section 2.4, respectively). Six and three larvae were included in each sample, respectively, with a total of three and five replicates, respectively.

### 2.8. Statistical Analysis

The experimental data were processed in Microsoft Excel, and graphs were created using the GraphPad Prism 9.0.0 software (San Diego, CA, USA). Statistical analyses were performed using the SPSS Statistics software (version 21.0; IBM, Armonk, NY, USA). Data were compared using an independent-samples *t*-test, with significance levels of * *p* < 0.05, ** *p* < 0.01, and *** *p* < 0.001.

## 3. Results

### 3.1. Effect of Bt Priming on RPW Development

**Figure 1:**
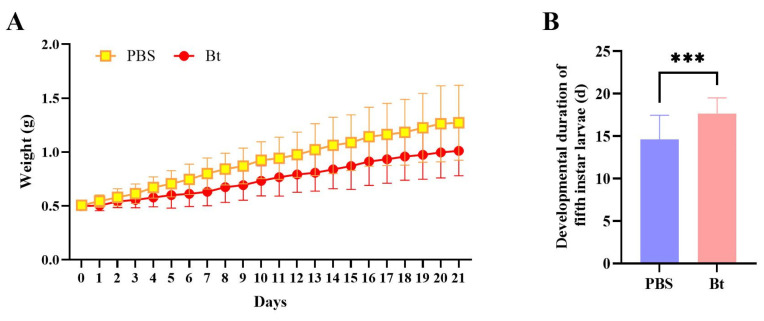
Biological parameters of the fifth-instar larvae of *Rhynchophorus ferrugineus* after Bt priming. (**A**) Weight gain of the fifth instar larvae after Bt priming. (**B**) Developmental duration of the fifth instar larvae after Bt priming. Data are shown as mean ± SE. Statistical significance was analyzed via independent-samples *t*-test. *** *p* < 0.001.

The development of fifth-instar RPW larvae was measured after Bt priming, which resulted in slower weight gain in RPW (Figure 1A). Compared with PBS, Bt priming resulted in a 7.6% reduction in body weight on the first day (*t* = 3.087, *df* = 58, *p* = 0.0031), and this difference gradually increased with time (Figure 1A; Table S2). The developmental time of the fifth-instar larvae with Bt priming was 17.67 d, which was significantly longer than that of the larvae injected with PBS, at 14.61 d (*t* = 3.815, *df* = 34, *p* = 0.0005) (Figure 1B). Therefore, Bt priming delayed the development of the RPW larvae.

### 3.2. Effects of Bt Priming on Hormone Contents

**Figure 2:**
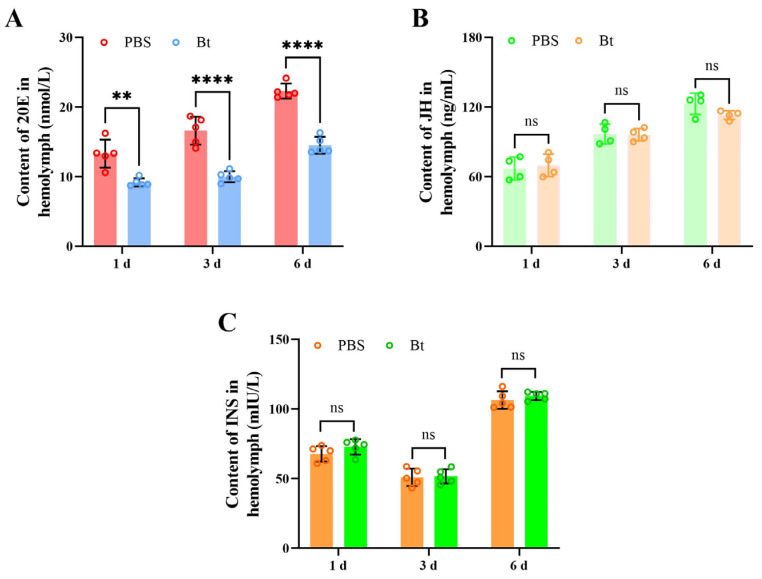
Contents of 20E, JH, and INS in the hemolymph of fifth-instar larvae of *Rhynchophorus ferrugineus* after Bt priming. 1, 3, and 6 d indicates the time after Bt priming. (**A**) Contents of 20E in the I think it’s OKhemolymph after Bt priming for 1, 3, and 6 d. (**B**) Contents of JH in the hemolymph after Bt priming for 1, 3, and 6 d. (**C**) Contents of INS in the hemolymph after Bt priming for 1, 3, and 6 d. Data are shown as mean ± SE. Statistical significance was analyzed by independent-samples *t*-test. ns: no significance (*p* > 0.05), ** *p* < 0.01, **** *p* < 0.0001.

The 20E, JH, and INS contents in the hemolymph of fifth-instar larvae were measured after Bt priming using ELISA. After Bt priming for 1, 3, and 6 d, the contents of 20E in the hemolymph significantly decreased by 31.0%, 39.8%, and 34.9% compared to the PBS control group, respectively (1 d: *t* = 4.423, *df* = 8, *p* = 0.0022; 3 d: *t* = 6.884, *df* = 8, *p* = 0.0001; 6 d: *t* = 10.78, *df* = 8, *p* = 0.0000) (Figure 2A). However, there were no significant differences in the contents of JH and INS between Bt and PBS priming at all time points (Figure 2B,C) (JH, 1 d: *t* = 0.3969, *df* = 6, *p* = 0.7052; 3 d: *t* = 0.1271, *df* = 6, *p* = 0.9030; 6 d: *t* = 0.1978, *df* = 6, *p* = 0.0953. INS, 1 d: *t* = 1.419, *df* = 6, *p* = 0.1935; 3 d: *t* = 0.1656, *df* = 8, *p* = 0.8726; 6 d: *t* = 0.9426, *df* = 8, *p* = 0.3735). Therefore, the reduction in the 20E titer may have inhibited RPW development.

### 3.3. Effects of Bt Priming on Transcription of 20E Synthesis Pathway Genes

**Figure 3:**
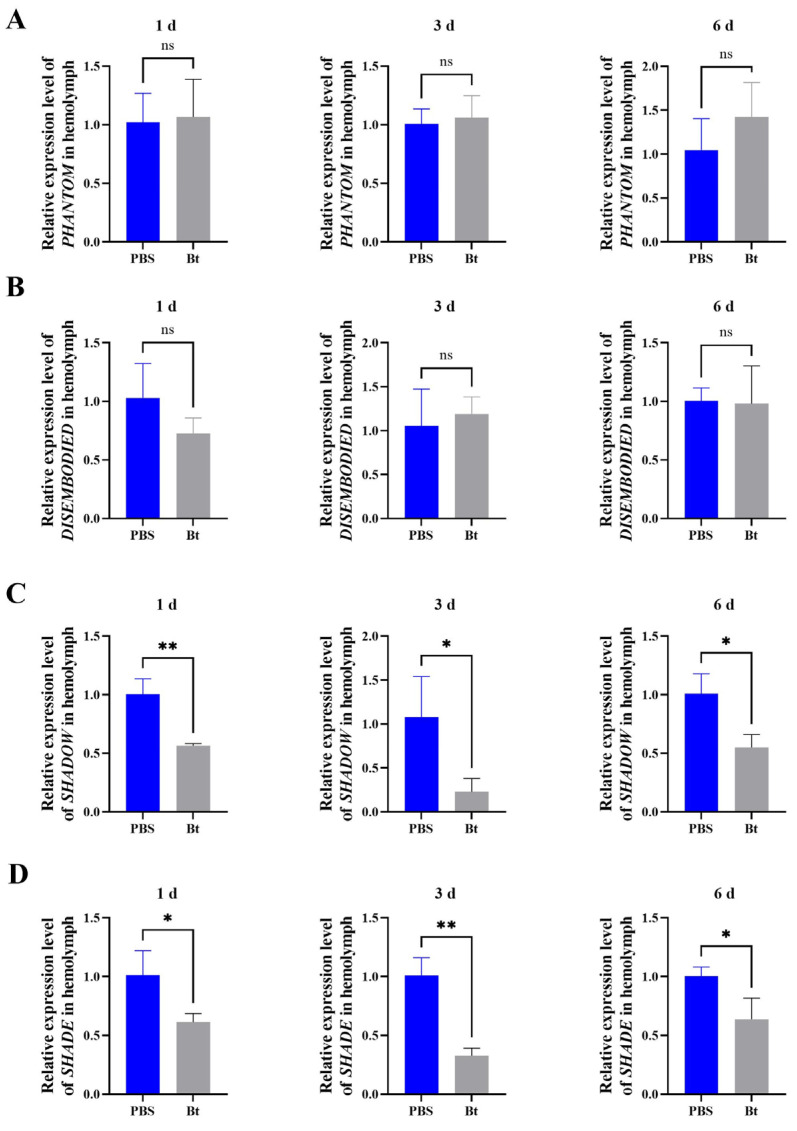
Relative expression of 20E synthesis genes in the hemolymph of the fifth-instar larvae of *Rhynchophorus ferrugineus* after Bt priming. (**A**) The relative expression of *PHANTOM* after Bt priming for 1, 3, and 6 d. (**B**) The relative expression of *DISEMBODIED* after Bt priming for 1, 3, and 6 d. (**C**) The relative expression of *SHADOW* after Bt priming for 1, 3, and 6 d. (**D**) The relative expression of *SHADE* after Bt priming for 1, 3, and 6 d. Data are shown as mean ± SE. Statistical significance was analyzed via independent-samples *t*-test. ns: no significance (*p* > 0.05), * *p* < 0.05, ** *p* < 0.01.

The expression of key genes in the 20E synthesis pathway in RPW was determined after Bt priming by RT-qPCR. There were no significant differences in the expressions of *PHANTOM* (1 d: *t* = 0.1986, *df* = 4, *p* = 0.8522; 3 d: *t* = 0.4032, *df* = 4, *p* = 0.7074; 6 d: *t* = 1.237, *df* = 4, *p* = 0.2836) and *DISEMBODIED* (1 d: *t* = 1.643, *df* = 4, *p* = 0.1756; 3 d: *t* = 0.5100, *df* = 4, *p* = 0.6369; 6 d: *t* = 0.1195, *df* = 4, *p* = 0.9106) between the Bt and PBS treatments at each time point (Figure 3A,B). However, the expression of *SHADOW* was significantly downregulated by 43.7%, 78.8%, and 45.4% at 1, 3, 6 d after Bt priming, respectively, compared to the PBS control group (1 d: *t* = 5.783, *df* = 4, *p* = 0.0044; 3 d: *t* = 3.000, *df* = 4, *p* = 0.0400; 6 d: *t* = 3.926, *df* = 4, *p* = 0.0172) (Figure 3C). The expression of *SHADE* significantly decreased by 39.6%, 67.4%, and 36.4% compared with the PBS treatment, respectively (1 d: *t* = 3.180, *df* = 4, *p* = 0.0335; 3 d: *t* = 7.140, *df* = 4, *p* = 0.0020; 6 d: *t* = 3.218, *df* = 4, *p* = 0.0324) (Figure 3D).

### 3.4. Effects of Bt Priming on Transcription of Toll Pathway Genes

**Figure 4:**
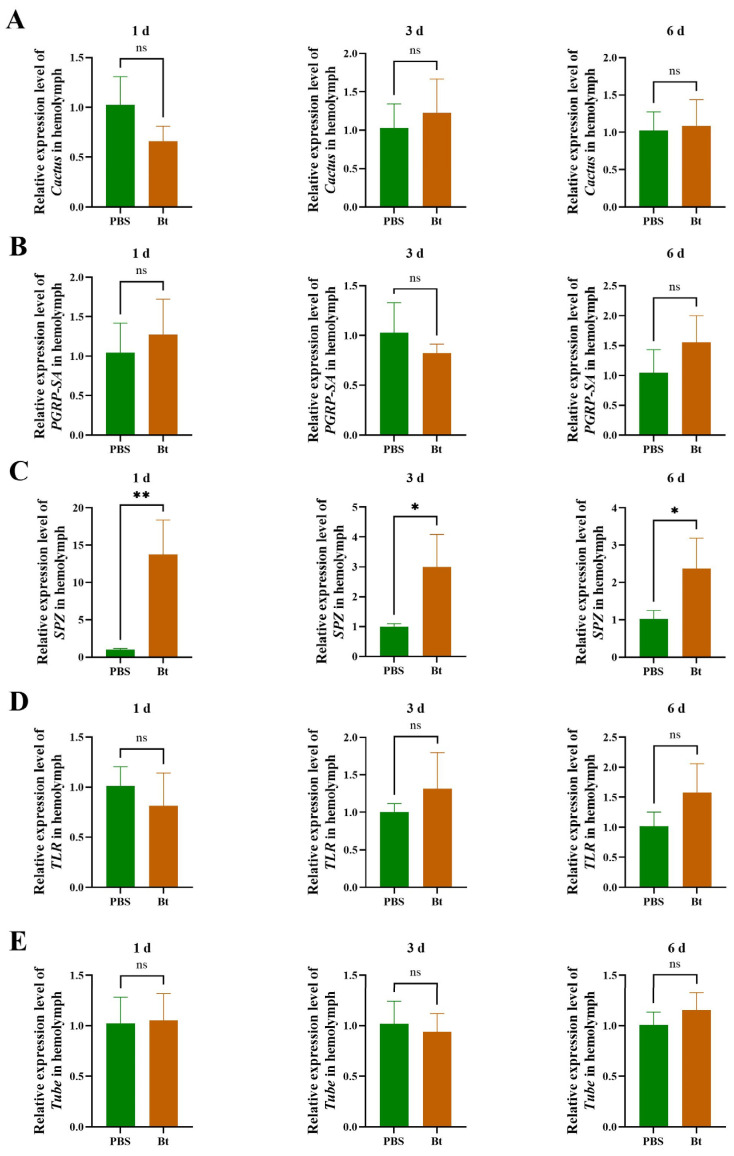
Relative expression of Toll pathway genes in the hemolymph of the fifth-instar larvae of *Rhynchophorus ferrugineus* after Bt priming. (**A**) The relative expression of *Cactus* after Bt priming for 1, 3, and 6 d. (**B**) The relative expression of *PGRP-SA* after Bt priming for 1, 3, and 6 d. (**C**) The relative expression of *SPZ* after Bt priming for 1, 3, and 6 d. (**D**) The relative expression of *TLR* after Bt priming for 1, 3, and 6 d. (**E**) The relative expression of *Tube* after Bt priming for 1, 3, and 6 d. Data are shown as mean ± SE. Statistical significance was analyzed via independent-samples *t*-test. ns: no significance (*p* > 0.05), * *p* < 0.05, ** *p* < 0.01.

The expression of key genes in the Toll pathway in RPW was determined by RT-qPCR after Bt priming. There were no significant differences in the expression of *Cactus* (1 d: *t* = 1.956, *df* = 4, *p* = 0.1222; 3 d: *t* = 0.6258, *df* = 4, *p* = 0.5654; 6 d: *t* = 0.2514, *df* = 4, *p* = 0.8139), *PGRP-SA* (1 d: *t* = 0.6862, *df* = 4, *p* = 0.5303; 3 d: *t* = 1.131, *df* = 4, *p* = 0.3214; 6 d: *t* = 1.511, *df* = 4, *p* = 0.2053), *TLR* (1 d: *t* = 0.9007, *df* = 4, *p* = 0.4187; 3 d: *t* = 1.087, *df* = 4, *p* = 0.3382; 6 d: *t* = 1.829, *df* = 4, *p* = 0.1415), and *Tube* (1 d: *t* = 0.1290, *df* = 4, *p* = 0.9036; 3 d: *t* = 0.4791, *df* = 4, *p* = 0.6569; 6 d: *t* = 1.188, *df* = 4, *p* = 0.3005) between the Bt and PBS treatments at each time point (Figure 4A,B,D,E). However, the expression of *SPZ* significantly increased by 13.6, 3.0, and 2.3 times at 1, 3, and 6 d after Bt priming, respectively, compared with the PBS control group (1 d: *t* = 4.786, *df* = 4, *p* = 0.0087; 3 d: *t* = 3.154, *df* = 4, *p* = 0.0344; 6 d: *t* = 2.801, *df* = 4, *p* = 0.0488) (Figure 4C). This result suggests that *SPZ* may represent a key node in the signaling pathway involved in Bt priming of RPW.

### 3.5. Effect of SPZ Silencing on SHADOW Expression and 20E Synthesis

**Figure 5:**
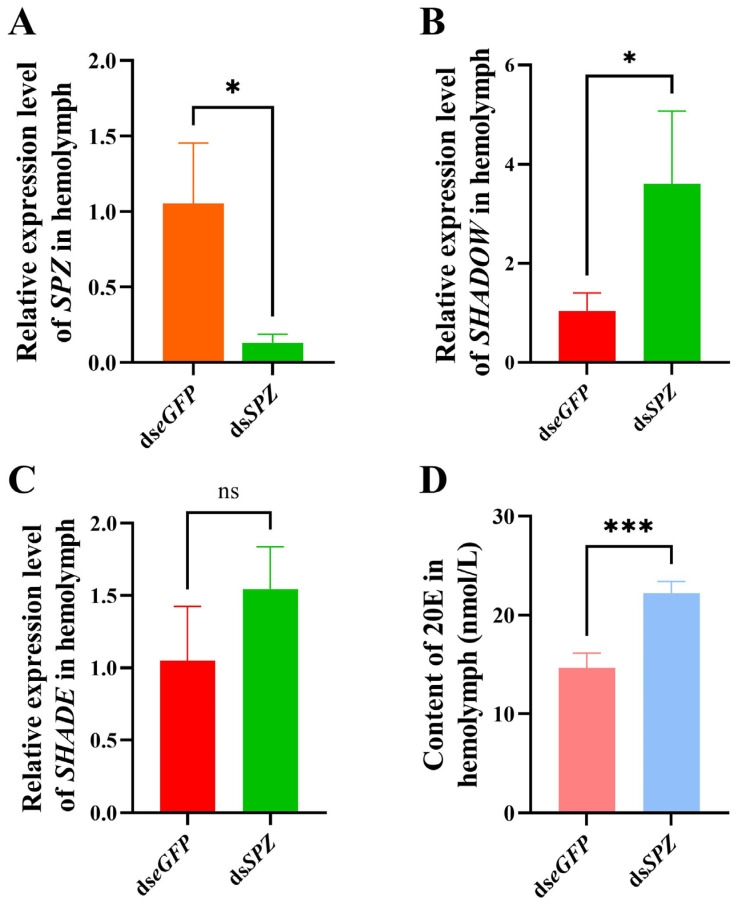
Effect of *SPZ* interference on *SHADOW* expression and 20E content in the hemolymph of the fifth-instar larvae of *Rhynchophorus ferrugineus*. (**A**) Interference efficiency of target genes *SPZ* at 24 h. (**B**) Relative expression of *SHADOW* following *SPZ* interference. (**C**) Relative expression of *SHADE* after *SPZ* interference. (**D**) The content of 20E in the PRW hemolymph following *SPZ* interference. Data are shown as mean ± SE. Statistical significance was analyzed via independent-samples *t*-test. ns: no significance (*p* > 0.05), * *p* < 0.05, *** *p* < 0.001.

After ds*SPZ* injection for 24 h, the mRNA expression level of *SPZ* in hemolymph was significantly suppressed by 87.6% compared to the ds*eGFP* control group (*t* = 3.957, *df* = 4, *p* = 0.0167) (Figure 5A), whereas the relative expression of *SHADOW* was significantly upregulated by 3.46 times compared with the control (*t* = 2.932, *df* = 4, *p* = 0.0427) (Figure 5B). However, there was no significant difference in the relative expression of *SHADE* between the ds*SPZ* and ds*eGFP* treatments (*t* = 1.801, *df* = 4, *p* = 0.1460) (Figure 5C). The contents of 20E in the hemolymph were significantly increased from 14.63 nmol/L for ds*eGFP* to 22.21 nmol/L for ds*SPZ* (*t* = 7.925, *df* = 6, *p* = 0.0002) (Figure 5D). These results indicated that *SPZ* inhibited 20E synthesis by negatively regulating the *SHADOW* expression in RPW.

### 3.6. SPZ Interference Led to Clearance of Bt Priming on Development

**Figure 6:**
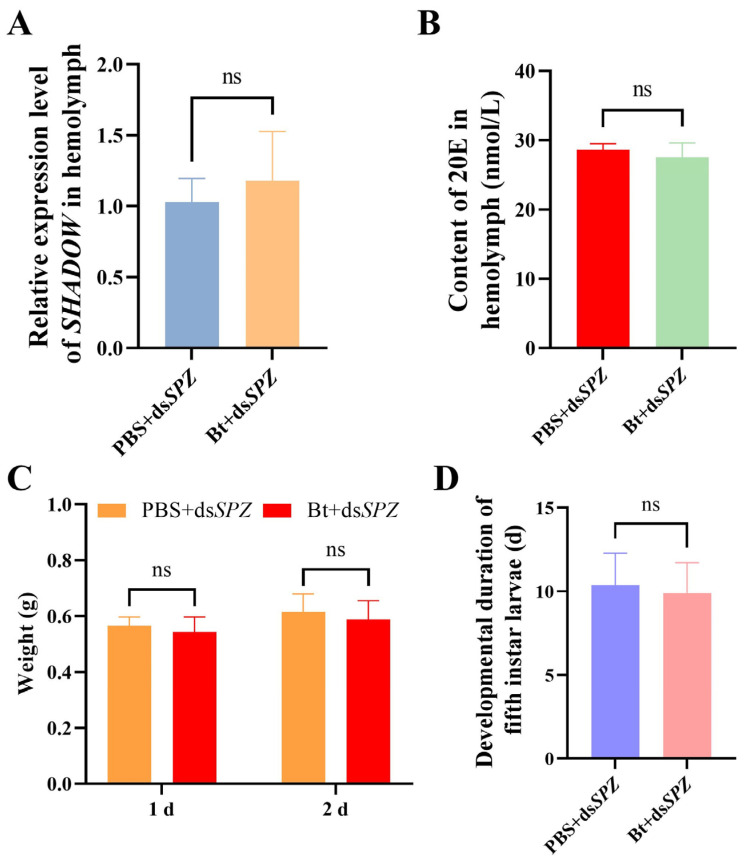
Effect of Bt priming on the fifth-instar larvae of *Rhynchophorus ferrugineus* after *SPZ* interference. (**A**) The relative expression of *SHADOW* via Bt priming after *SPZ* interference. (**B**) The content of 20E in the PRW hemolymph by Bt priming following *SPZ* interference. (**C**) Weight of the fifth-instar larvae of PRW following 1 and 2 days of Bt priming after *SPZ* interference. (**D**) Developmental duration of the fifth-instar larvae of PRW via Bt priming after *SPZ* interference. Data are shown as mean ± SE. Statistical significance was analyzed via independent-samples *t*-test. ns: no significance (*p* > 0.05).

The inhibitory effects of Bt priming were reversed when *SPZ* was silenced in RPW. There was no significant difference in the *SHADOW* expression from PBS and Bt treatments following inhibition of the *SPZ* expression (*t* = 0.3900, *df* = 4, *p* = 0.7149), as well as in the 20E synthesis (28.67 nmol/L for PBS and 27.53 nmol/L for Bt) (*t* = 1.132, *df* = 8, *p* = 0.2904) (Figure 6A,B). Additionally, there were no significant differences in weight on the 1 and 2 days (1 d: *t* = 1.381, *df* = 28, *p* = 0.1783; 2 d: *t* = 1.167, *df* = 28, *p* = 0.2529) and developmental duration (10.36 d for PBS and 9.91 d for Bt) (*t* = 0.5720, *df* = 20, *p* = 0.5737) of the fifth-instar larvae via Bt and PBS priming after inhibition of *SPZ* expression in RPW (Figure 6C,D).

## 4. Discussion

A growing number of contemporary studies have shown that an early microbial infection or parasitic attack can enhance the immune response of invertebrates through immune priming to subsequent exposure to pathogens or parasitoids [6,26,45,46,47], and this effect can even be passed from parents to offspring [6,48]. Previous studies have confirmed that pathogenic infections, such as Bt, *Escherichia coli*, *Serratia marcescens*, and *Metarhizium anisopliae*, can also cause the immune priming of RPW [7,12]. Immune priming primarily requires changes in PO activity, antibacterial activity, hemocyte differentiation, phagocytosis, and the expression of immune-related genes [7,12]; however, immune priming is an energy-consuming process, and its trade-offs with fitness are poorly understood. Our study analyzed the fitness cost of Bt priming in RPW and the trade-offs between development and immunity.

We observed that the body mass of immune-primed larvae increased more slowly, and the developmental period was significantly prolonged compared with that of the PBS control. Our results support previous studies showing that the activation of the immune response delays growth and development in the host [47]. Microbial infections can induce changes in host hormone levels, thereby regulating host development [35,49,50]. For example, an entomopoxvirus infection inhibited 20E synthesis, thus prolonging the developmental period of *M. separata* larvae [35], and a microsporidian infection prevented the development of *Lacanobia oleracea* by increasing the levels of JH [51]. Similar results were observed in our study, where Bt priming resulted in a reduced 20E content and slower development in the fifth-instar larvae of RPW. Simultaneously, Bt priming significantly downregulated the expressions of *SHADOW* and *SHADE* in the 20E synthesis pathway. *SHADOW* normally produces 2-deoxyecdysone to catalyze the generation of ecdysone, whereas *SHADE* catalyzes the hydroxylation of ecdysone to 20-hydroxyecdysone [52]. A previous knockdown of *SHADOW* reduced the 20E titer and prolonged the developmental duration of *Plutella xylostella* [53], and inhibition of *SHADE* transcription also blocked 20E synthesis, increased mortality, and prolonged development in *Laodelphax striatellus* nymphs [54]. These results indicate that Bt priming regulates RPW development by controlling the 20E synthesis pathway, resulting in a fitness cost.

Immune priming activates a series of molecular responses that inhibit the proliferation of viruses, parasites, and bacteria in arthropods [45]. Wu et al. showed that Bt priming upregulated the expression of immune-related genes, *cecropin*, *hemolin*, *gallerimycin*, and *lysozyme1*, in *Galleria mellonella* [47]. The process of insect immunity begins when host cells precisely recognize and bind to biological macromolecules with pathogen-associated molecular patterns through pattern recognition receptors, ultimately stimulating immune signaling regulatory networks [55,56,57]; the most important of these networks are the Toll and IMD signaling pathways [3]. Different immune response pathways participate in combating various pathogenic infections [3,58]. For example, the Toll pathway mainly influences fungal and gram-positive infections (e.g., *B. thuringiensis*), whereas the IMD pathway primarily addresses gram-negative infections (e.g., *S. marcescens*) [59]. In this study, the expression of the *Spätzle* (*SPZ*) gene in the Toll pathway significantly increased after Bt priming. *SPZ* binds to Toll receptors as a dimeric ligand, initiating the secretion of antimicrobial peptides by activating immune signaling pathways [3]. Valanne et al. also verified that *SPZ* is an extracellular ligand for Toll receptors and plays a crucial role in activating antimicrobial peptides [60]. Muhammad et al. reported that silencing *SPZ* significantly downregulated the expression of antimicrobial peptide genes, *RfColeoptericin* and *RfCecropin*, resulting in increased mortality of RPW larvae caused by Bt infection [61]. We conclude that *SPZ* strongly induced the immune response by Bt priming in RPWs.

The maintenance and activation of the immune response induced by immune priming enhance the host’s antimicrobial ability but also cause a negative impact because of the high energetic cost of immunity [62,63,64,65]. Life history theory posits that organisms allocate their limited energy to different biological functions through trade-offs [66]. Individuals investing in survival maintenance, including pathogen resistance, may have less energy to devote to other biological functions, thereby reducing their fitness for development and reproduction [67,68,69]. Contreras-Garduño et al. determined that *Plasmodium berghei* priming more effectively controlled a challenge from the same parasite and maintained a higher survival rate in *Anopheles albimanus*; however, this increased effectiveness was accompanied by the inability to lay eggs [70], thus demonstrating a trade-off between immune priming and reproduction.

## 5. Conclusions

We demonstrated that Bt priming activated immune resistance via *SPZ* but inhibited development by regulating 20E synthesis; this result suggests that Bt priming induces a fitness trade-off between immunity and RPW development. Our recovery experiments showed that a knockdown of *SPZ* reduced the fitness cost caused by Bt priming, indicating that *SPZ* is a key regulator of developmental and immune trade-offs induced by Bt priming in RPW. These findings highlight the co-effects of immune, hormonal, and developmental factors on the fitness trade-offs of RPW, thereby more comprehensively detailing the evolution of immune defenses in insect pests and providing new insights and targets for RPW biological controls.

## Data Availability

All data included in this study are available upon request by contact with the corresponding author.

## Supplementary Materials

The following supporting information can be downloaded at: https://www.mdpi.com/article/10.3390/insects15120925/s1, Table S1: Primers for qPCR; Table S2: Statistical analysis of body weight of the fifth instar larvae of *Rhynchophorus ferrugineus* after Bt priming.
