# Supplementary material for: Spätzle Regulates Developmental and Immune Trade-Offs Induced by Bacillus thuringiensis Priming in Rhynchophorus ferrugineus
Source: Insects. 2024 Nov 26;15(12):925. doi: 10.3390/insects15120925 (PMC11677516; doi:10.3390/insects15120925)
Supplement: Supplementary file 1 [file insects-15-00925-s001.zip › insects-3298405-supplementary.pdf]

Table S.1 Primers for qPCR

Primer for qPCR	Secquence(5' - 3')
GAPDH-F	CCAAGGGAGCCAAGCAATT
GAPDH-R	CGCTGATGCCCCTATGTATGT
PHANTOM-F	TACCGTTTCTCGATCCCAAG
PHANTOM-R	CCTGTCGTTACGTCAAGCAA
DISEMBODIED-F	AACCAGAACGGTGGTTGAAG
DISEMBODIED-R	ACAAGATCTGGGACCGTGAC
SHADOW-F	CAGGTTCCACACCGAAACTT
SHADOW-R	TACATGGCACTGGTCCGATA
SHADE-F	AAGGTGGCTGAACGAAGAGA
SHADE-R	TCCCGGACATATTCTTCTGC
PGRP-SA-F	TACTGACTCGTTCGCAGTGG
PGRP-SA-R	CCATCTCCTCCGATCAAAAA
SPZ-F	ACCCGTGTACATTCCAAAGC
SPZ-R	TAGTTGGATTGCACGAGCTG
TLR-F	CAAAGCGTGACAAAACTGGA
TLR-R	ACAGCGTCTTTCGGCTTTTA
Tube-F	CGCCGAGTATTACACAACGA
Tube-R	GAGCCCTCTCAATGAACCTG
Cactus-F	CCAAACACGTAAATGCGATG
Cactus-R	TTACATCCGCTCCGTAGCTT
dseGFP-F	taatacgactcactatagggCAGTGCTTCAGCCGCTAC
dseGFP-R	taatacgactcactatagggGTTCACCTGCCGTTCTTGA
dsSPZ-F	taatacgactcactatagggCAGCTCGTGCAATCCAACTA
dsSPZ-R	taatacgactcactatagggGATTCGGGCATATTCACCAC

Table S.2 Statistical analysis of body weight of the fifth instar larvae of *Rhynchophorus ferrugineus* after Bt priming

Days	t Ratio	DF	P value
1d	3.087	58.00	0.0031**
2d	1.858	58.00	0.0389*
3d	2.696	58.00	0.0092**
4d	3.835	58.00	0.0003***
5d	3.370	58.00	0.0013**
6d	3.983	58.00	0.0002***
7d	4.814	58.00	0.0000****
8d	4.484	58.00	0.0000****
9d	4.475	58.00	0.0000****
10d	4.724	58.00	0.0000****
11d	3.641	58.00	0.0006***
12d	3.780	58.00	0.0004***

13d	3.978	58.00	0.0002***
14d	3.844	58.00	0.0003***
15d	3.545	58.00	0.0008***
16d	3.567	58.00	0.0007***
17d	3.454	58.00	0.0010**
18d	3.293	58.00	0.0017**
19d	3.495	58.00	0.0009***
20d	3.428	58.00	0.0011**
21d	3.412	58.00	0.0011**

Note: ns indicates no significant difference, *$P<0.05$, **$P<0.01$, ***$P<0.001$, ****$P<0.0001$ indicates statistically significant difference.
